# Systematic survey on data security in wireless body area networks in IoT healthcare system

**DOI:** 10.3389/fmed.2024.1422911

**Published:** 2024-07-30

**Authors:** Wang Jian, Alia Tabassum, Jian Ping Li

**Affiliations:** ^1^School of Artificial Intelligence, Neijiang Normal University, Neijiang, Sichuan, China; ^2^Department of Computer Science and Software Engineering International Islamic University Islamabad, Islamabad, Pakistan; ^3^School of Computer Science and Engineering, University of Electronic Science and Technology of China, Chengdu, China

**Keywords:** WBAN, data security, encryption decryption, SLR, healthcare

## Abstract

In the Internet of Things (IoT) healthcare sector, the wireless body area network (WBAN) is being used to optimize medical results by tracking and treating patients as they go about their daily lives. Health insurance has also been one of the cybercriminal's main goals. The Systematic Review of IoT Healthcare systems particularly wireless body area networks is significant, to reach the benefits and challenges faced by existing methods in the domain. This study provides a systematic survey of WBAN data protection. Various types of devices are used in medical science to detect and diagnose diseases. The network is an integral part of medical science in today's era. In medical sciences, sensors take data from a problematic place like cancerous cells. This research discussed a lot of techniques in the literature review. Most of them are not able to fulfill the requirements. If an unauthorized person reaches the data that can be a severe issue, like the diagnosed disease was blood cancer, and after unauthorized access manipulation can change even the diagnosed issue in the database. A doctor can prescribe the medication based on provided data that has been manipulated by unauthorized persons. Several existing schemes are explored in the literature to determine how the protection of sharing patients' healthcare data can be improved. The systematic literature review (SLR) of multiple security schemes for WBAN is presented in this survey paper.

## 1 Introduction

The security of WBAN (1) is essential and should not be forgotten. It is confidential, sensitive medical information and must be shielded from unauthorized persons who can use details that may be harmful to the person (2). By using WBAN with the use of various varieties of sensors to track the patients to detect any life-threatening diseases healthcare applications are enhanced. This technology aims to increase the quality of medical services delivered and reduce certain related costs. BAN has a broad spectrum of applications, like tracking the medical conditions of patients and optimizing their response to clinical guidelines, but protection and privacy are some of the main concerns in BAN-based healthcare systems at the same time medical data must be kept protected from risk factors and hackers during storage and transmission (3, 4). The existing literature discusses data privacy and protection (5, 6), but it doesn't go into depth about the SLR and the requirements for collecting data. There is a lot of literature on security strategies, but it isn't focused on security research. This study used three different Databases including IEEE, ACM, and Springers. The existing literature discusses data privacy and protection, but it doesn't go into depth about the SLR and the requirements for collecting data. There is a lot of literature on security strategies, but it isn't focused on security research. This study used three different Databases including IEEE, ACM, and Springers. Develop a string by using the objective of all papers and then used 3 synonyms of each keyword in the string. This research discussed inclusion criteria in which we have two parts one is included and another is excluded (not included). The thesis, newspaper, books and are not included in the inclusion criteria and title-based, abstract-based, and objective-based research papers are included. After that design, this study performs objective-based filtering and abstract-based filtering. Aim objectives and methodologies of each paper are discussed below. And also provide critical analysis. The conclusion of this research is to provide an efficient way for data security in WBAN. Privacy in WBAN is important and should not be forgotten. Medical data is important and must be shielded from unauthorized access. The motivation is to compile and research papers that deal with security issues in depth. In this research papers were identified after an extensive search using strings in different databases. The papers were then screened using title and abstract-based evaluations to determine if the study was appropriate or not. We present the comparative analysis of data in tabular form in this section. The study concluded that WBAN is a more effective approach to exchanging data between doctors and patients by doing this survey. In Table 1, existing surveys on data security in WBAN are discussed. This shows the strength of this survey paper with already existing survey papers using the comparison method. Compare all survey papers in terms of communication cost, energy consumption, storage, etc.

**Table 1:** Overview of existing research.

**References**	**Year**	**Objective**	**Techniques**	**Criticism**
Rehman et al. (7)	2020	The specification for storage, the cost of computing and communication, time, and cost computational.	Internet protection protocol and automatic validation (AVISPA) platform.	The lack of usability features, and different routing threats, like a privileged insider, user, and server impersonation, do not provide an effective password change point.
Jabeen et al. (8)	2020	Complexity of time, key generation development, computational time algorithm for encryption.	Novel data protection genetic-based encryption scheme, AES.	AES also has disadvantages, like high cost of calculation for hardware, large in size, use of CPUs.
Hasan et al. (9)	2020	Reliability, Trust, provide less cost and time.	-	Execute more slowly, The more complicated mathematical model.
Jabeen et al. (8)	2020	The study examines a variety of current strategies to determine how patient health data protection can be improved.	-	
Parvez et al. (10)	2019	Electronic health records (EHR) systems handle the internet's most private information, improve security.	Electronic Healthcare Repository (EHR).	Changes in workflow temporary loss of productivity.
Chowdhury et al. (11)	2018	Portable support in the form of consuming minimum energy consumption creates a larger framework for a PMS end-to-end safe communication way for different medications.	AES, MQTT	AES also has disadvantages, like high costs of computation, and high specification for hardware. More difficult, wide footprint, use of CPUs, high storage and power.
Shanthapriya and Vaithianathan (12)	2018	Integrity, reliability, medical privacy, and security.	The polynomial curve and the Steganography technique are generated.	The key concept behind these strategies is that without being found, edges will bear more variation than smooth areas.
Braham et al. (4)	2018	The energy-efficient system, energy-efficient protection protocol, reducing the cost of healthcare.	-	No formal syntax or semantic logic is restricted to authentication protocol analysis. Does not have proper encryption accounts.
Malik et al. (13)	2018	Resolving variously defined gaps in storage requirements, enhancing privacy, improving security.	Internet protection protocol and application automatic validation (AVISPA) platform.	Lack of functionality features various known attacks, such as privileged insider, user, and server impersonation, do not include.
Anwar et al. (14)	2018	-	AES and MQTT	Theoretically, file-type attacks are successful DOS, IoT attacks are not suitable for high-complexity sensor networks.
Roy et al. (15)	2017	-	AES, ECC	The implementation of AES and ECC is challenging. Complex, time-consuming, and hard to execute.
Hasan et al. (9)	2020	Achieve high throughputs and low latncy for emergency traffic.	SDN (Software-defined Networking)	Vulnerabilities in security, inconsistency SDESW's flow demands rise as the network becomes more complex.
Ren et al. (16)	2019	only the designated person has access to the user' data.	DVSSA	Complexity and Time Consumption.
Zhang and Ma (17)	2018	Improve user privacy at a low cost.	A security mechanism that is aware of the channel	It takes a long time to authenticate each node.
Shanmugavadivel et al. (18)	2021	In a cloud environment improve data security using the AES.	AES, Genetic Algorithm for Task Flow Scheduling.	High complexity.
Singh and Prasad (19)	2021	Provide detailed study of different systems and protocols for dealing with energy efficiency and security.	-	
Soni and Singh (20)	2021	Lower execution cost, calculation time, and power consumption when compared to other protocols.	LAKA Lightweight Authentication and Key Agreement Protocol.	Higher power consumption.
Sandhu and Malik (21)	2020	The goal of this article is to efficiently transmit data based on the priority of the data.	PAP(Priority aware protocol)	As the priority level rises, so does the amount of energy expended.
Li et al. (22)	2017	The goal of this article is to use physical channel information to eliminate the need for additional hardware requirements.	RSSI(Received Signal Strength Indicator).	An algorithm is difficult to implement because it is complex.

### 1.1 Motivation of the study

The existing literature discusses data privacy and protection, but it doesn't go into depth about the SLR and the requirements for collecting data. Many methods for improving technical efficiency have already been established in this area, but current work required more accuracy. Another relevant and high-quality SLR survey has been rationalized, but it used a limited amount of established literature, which could impact methodology comparisons. Additionally, a systematic analysis is based on comparing and highlighting study gaps; however, this survey does not include many details regarding current WBAN literature schemes. The research conducts a systematic literature review, which is used to support the proposed SLR in the survey. The major contributions of this study are as follows:

1. To create a taxonomy that covers the security encryption techniques that are required in the WBAN setting. Existing work has been addressed in depth in each section of the taxonomy to address a variety of issues, including time, cost, and predicting network attacks.
2. From 2017 to 2024, we followed the Preferred Reporting Items for Systematic Reviews and Meta-Analyses (RISMA) flow chart to search the literature, delete duplicate information, screen, exclude, and include articles.
3. SLR can be used for very relevant schemes that concentrate on protecting healthcare data by preventing security threats while using less memory.
4. To encourage researchers to provide effective solutions to problems, a security review with criticism is performed.

The rest of the paper is arranged as follows. Section 1 presents the introduction to WBAN. Section 2 shows the Systematic literature review (SLR). Section 3 shows the Detailed Literature Review and Section 4 concludes this work.

## 2 Systematic literature review

This study chose a year range(2017–2024), selected three synonyms for each string keyword, searched three databases (ACM, Springer, IEEE), and then conducted random searches against strings. This research created a string containing all of the papers' objectives and then utilized three synonyms for each keyword in the string. Then this research talked about inclusion criteria, where one component is included and the other is excluded (not included). The thesis, newspaper, books and are not included in the inclusion criteria, and title-based, abstract-based, and objective-based research papers are included.

### 2.1 Research selection procedure

**Figure 1:**
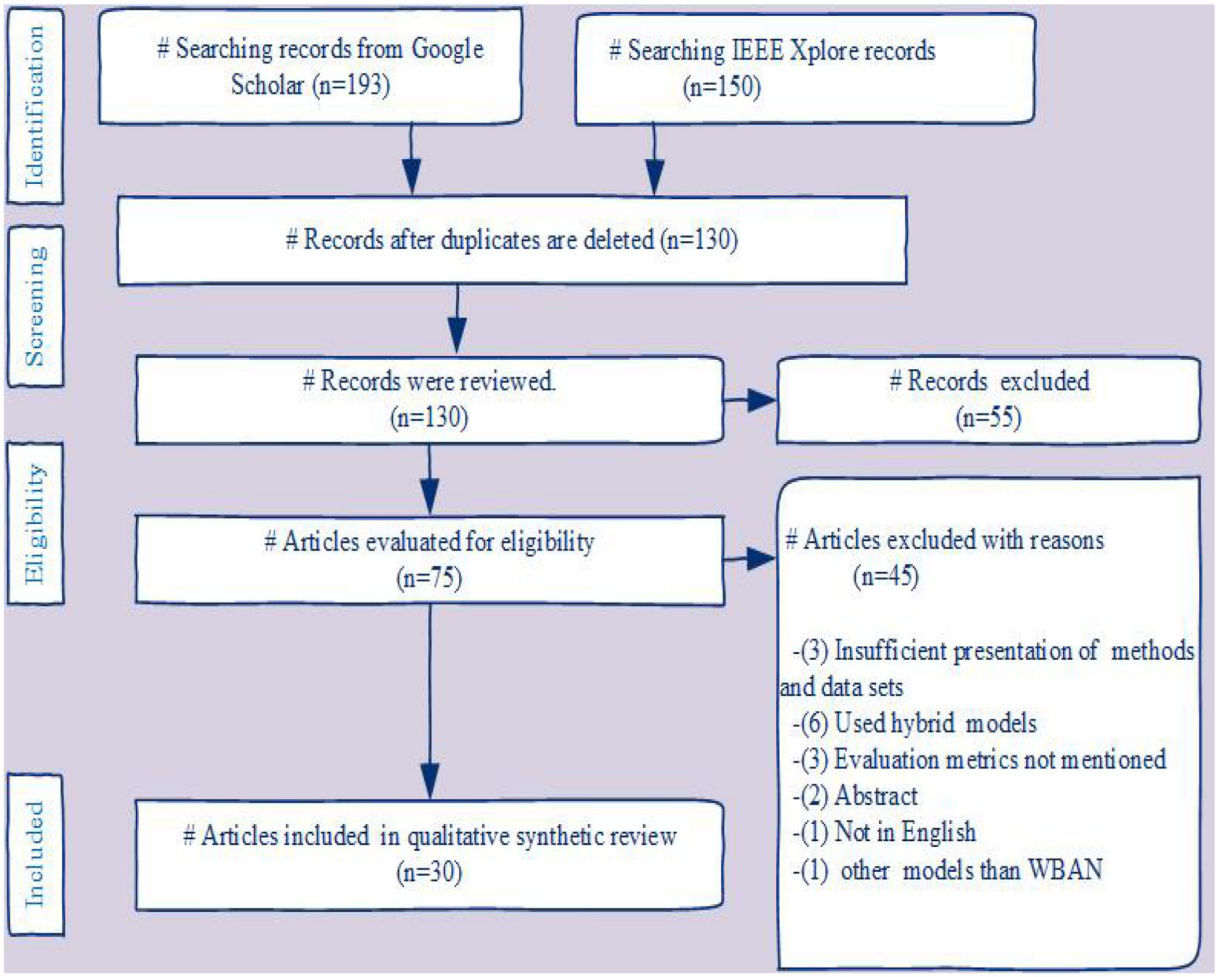
The PRISMA flow diagram depicts the procedure by which we searched the literature, removed duplicate records, screened, excluded, and included articles in our systematic review.

The PRISMA (23) flow chart in Figure 1 demonstrates our survey's systematic review procedure. In the selection process, research papers from the years 2017 to 2024 are included. Currently, 130 papers are being considered. publications that fulfill the study criteria are selected after searching for similar publications in various databases. During an initial review, 75 papers were shortlisted, and 30 relevant articles that met the requirements were included in the survey.

#### 2.1.1 Detailed literature review

WBAN is a multifaceted network that includes a variety of sensor hubs that track and relay data in real-time in a variety of situations. Sensor nodes collect vital information and send it to a medical server for further analysis. Since data includes highly confidential and important patient information, data security and safety is a critical challenge. WBAN information security is being investigated over a long period, from 2017 to 2024. This research literature focuses on various schemes such as SHA (Secure Hashing Algorithm), AES(Advanced Encryption Standards), and many others.

#### 2.1.2 AES based schemes

This research paper's (11, 24) goal is to build and apply a safe end-to-end PMS by focusing on the secure wireless connection gateway sensors with a lightweight encryption protocol that consumes minimal power. The research goal should be to provide protection and authorization processes to ensure that during the entire communication route, the data is not disclosed to an external observer nor damaged by a malicious sensor inside or in the vicinity of the WBAN. Lightweight encryption protocol, low energy consumption, a wider system for different medications, and an end-to-end safe communication network for a PMS are some of the key objectives of this study. The wireless body area network (WBAN) which is also used to capture the sensitive medical information of the patients is the access network of the users through a server in which the data of the patients is processed. In comparison to the literature approaches, the research work (8) aims to have less computational time complexity and a cost-effective genetic-based algorithm. This method also introduces a new algorithm for a key generation that has fewer steps and fewer computational methods. After generating the patient's data, the genetic-based lightweight encryption algorithm was applied over the nanosensors units. Genetic-based light encryption algorithm applied after producing the data of patients and over nanosensors devices. The encrypted information is then transmitted to the server, which further transforms it through a wireless network. Patients can also be tested with remote medical nanosensors nowadays, also for the collection of ongoing patients records, WBAN includes connected small sensors that are distributed via the networks for further processing. Cloud-based WBAN has recently gotten a lot of interest, but the cloud has many disadvantages in terms of data management and security. Consider these issues using the Advanced Encryption Standard (AES) and the Genetic Algorithm, this (Shanmugavadive) research provides improved data security and efficient task flow scheduling (GA). WBAN should address two critical criteria to deliver reliable services data security and privacy. Fake data and information in medical records can lead to major problems. If a person alters the values of gathered information and the physician prescribes medication based on the changed information, significant health problems and even death might occur (18).

#### 2.1.3 Data authentication

An essential component of Wireless Body Area Networks (WBAN) security and privacy protocols is the authentication of sensitive health-related data transmitted through the network. It is essential that both WBAN nodes have data authentication, and the coordinator must be able to confirm that the data is being sent from a reliable source and not a fraudster. Symmetric approaches, which generate the MAC (Message Authentication Code) of the whole set of data using a shared key, are used to verify the source of the data (25).

#### 2.1.4 Data authorization

A user's identity, role, or permissions determine which data or resources they can access or cannot access. This process is known as data authorization. It involves verifying users' identities and figuring out if they are authorized to view, edit, or remove data (25).

#### 2.1.5 Block cipher based schemes

This research work (7) aims to defend from various known cyberattacks, in particular, the vulnerability attack also on the base station and the dos attacks on the sensor node. These research findings and safety review show that in terms of storage needs, computing, and communication costs, the suggested improved system has overcome various established gaps. The goal of this paper is to establish a framework for safeguarding patients' health data from all safety difficulties. Requirements for storage, cost of computing and connectivity, time, and cost of computing. The suggested security system demonstrates its effectiveness in protecting against various known cyber-attacks, especially the compromise attack on the base station and the doc attacks on the sensor node. This paper's (15) goal is to propose a cost-effective framework that prevents unauthorized attackers from removing data packets or forwarding false data. This paper's goal is to present novel data protection mechanisms for WBAN that are capable of detecting getting into trouble relay nodes or links. The process refers to the routing algorithm for AOVD. The non-homogenous pattern of Poisson is used here to describe the possibility of malicious actions. The protection does not add any new packets of controls. To access performance, SLR on AODV is simulated and the results are compared with AODV. At a low cost, it is used to detect harmful intruders. The wireless body area networks (WBAN) are common options for a wide variety of health, sports activities, and recovery current study applications. In providing secure identification using an encryption mechanism, some existing WBAN routing protocols can be found, but they do not provide a lightweight communication solution. An energy-efficient framework is proposed in this paper that stops unauthorized intruders by dropping data packets or forwarding fake data. While it can communicate with any other reactive WBAN routing algorithm, the algorithm can be applied on the Adhoc On-demand distance-vector machine (AODV) protocol. In detecting malicious nodes with minimal latency, the protocol is simulated and results show its effectiveness.

#### 2.1.6 ZigBee

This research paper (13) first gives an overview of WBAN, how it was used for medical surveillance, then highlights its design, significant security, and privacy specifications, and attacks on specific network layers in a WBAN, and finally talks about different encryption protocols and laws to provide WBAN data protection solutions. Provides WBAN protections sensors are used to capture a patient's confidential and valuable medical data, are they may even be used in sports. WBANs connect with the device and other applications such as ZigBee, WI-FI, cellular networks, and applications for the wireless personal area network (WPAN). The wireless body area network is a series of wireless sensors that can be mounted in or out of the body of the human or living person, thus detecting or tracking the body's functions and adjacent circumstances.

#### 2.1.7 BAN detection

This paper (4) aims to review BAN communication standards, security risks, and BAN-based applications weaknesses, as well as current privacy and security processes. Privacy and security problems and the internet technology used in a BAN are outlined in the report. This technology aims to increase the quality of medical services rendered and reduce certain related costs. BAN has a wide variety of uses, such as tracking the health conditions of patients and optimizing the response to treatment plans, but protection and safety are among the main concerns in BAN-based healthcare systems at the same time, as medical data must be kept protected from adverse reactions and threats during stroke and transmission. Reducing healthcare cost, and energy-efficient climate, protocols for energy-efficient protection. Many studies have shown that if diseases are identified in their initial phases, there is a way to detect them.

#### 2.1.8 Hashing algorithm

This paper (14) aims to design Safe hashing algorithms (SHA) and encryption techniques used in research reviews to make data transfer more secure and efficient (14). It creates digital signatures using a hash method to move patient data more stably and authentically. This proposed algorithm makes use of an asymmetric key generation technique, which uses a pair of public and private keys, making the algorithms slow and more complex. Protecting Data Communication in WBAN through Digital Signatures, the proposed technique is based on a combination of different methods for securing data in WBAN by using protected keys and digital signatures. BNC digitally signs each data packet to SK and sends it to all sensor nodes in the network. WBAN (Wireless Body Area Network) is a special form of sensor network that connects patients with medical service providers via the Internet to exchange crucial health data. WBAN offers several advantages, including location-independent monitoring, no influence on patients' movement, early illness diagnosis and prevention, remote patient support, and so on. To ensure security, researchers have proposed several health data transmission techniques. The author (Soni) proposes a low-cost health authentication and key agreement technique that is both secure and lightweight. The suggested protocol uses a one-way hashing algorithm (SHA-256), and the National Institute of Standards and Technology (NIST) has determined that it is safe against the polynomial-time method (20).

#### 2.1.9 Multiple scheme

Mehmood et al. (26) aims to design a framework for the portable authentication process and session key arrangement between sensor nodes and health professionals that discuss both patterns of communication. The safety review shows that required security features are maintained. The purpose of this paper is to implement lightweight user security mechanisms that facilitate internal and external information exchange to build a safe session key between a health professional and a particular sensor node linked to the body of the patient. In the future, the scheme will be applied in an actual system in which the sensor nodes mostly on the patient's body communicate with mobile devices, cloud services, authentic gateway, and health professionals. The wireless body area network (WBAN) is also an IOT-based health service that greatly improves health treatment by allowing patients' health conditions to be tracked remotely. This paper (12) aim is an attempt to examine that IOT based WBAN security infrastructure on a base of the main security agreement scheme. Key encryption techniques are extremely inefficient in terms of computing, processing, and energy usage. In tier 1 of WBAN, this paper mainly focuses on various primary agreement frameworks. Four different groups separate the private key agreement schemes, conventional key framework, physiological key strategy, hybrid scheme, and private key agreement strategy. The Internet of things (IoT) (27) is one of the newest technologies these days that has consumed a lot of possibilities. Wireless body area network (WBAN) also is such emerging field that provides a remote ability to prevent and collect patients' health data using IoT based wearable biosensors. In IoT devices that are extremely resources constraints, their architectures are discovered to be ineffective. This study is an attempt to examine the IoT-based. The goal of this paper (12) is to create a polynomial-based curve for a safe system that helps the patient with dignity, authenticity, confidentiality, and privacy. An attacker can access the medical data of the patients that are stored in the controller or hack the data while communicating through wireless communication, without any of the patient's permission. An attacker can alter the message produced within the BAN before they are transmitted to a receiver (such as location, layout, quality, query, etc.) or change the communication content being transferred from the BAN to an external entity(e.g doctor). Farooq et al. (28) proposed a method to secure physical layer (PHY) transmission. This approach encrypts data without requiring the keys. Physical Layer Security The sensor nodes in multi-hop WBAN use the MTFG (Multi-Hop Topology Formation Game) algorithm to create a spanning tree for multi-hop communication in the uplink of the WBAN. This algorithm can be implemented in a distributed manner, among each sensor being aware of the presence of its neighbors to choose the best direction. The system's performance is evaluated in a variety of situations, and the results show that the suggested scheme has the best performance, which can be tailored to meet the competing needs of protection and latency for different applications. This article offers software-defined networking (SDN)-based WBAN (SDWBAN) architecture for application-specific traffic control to address these challenges. The suggested system achieves high throughput and low latency for emergency traffic in SDWBANs, according to the results of the paper's experiments. The objective of this paper is a scalable and adaptable SDWBAN framework that allows for dynamic network control as the number of apps on the network grows (traffic management) (9). WBAN is a sensor network with nodes that may be attached outside or within the body. Priority aware protocol (PAP) was proposed in this (Sandhu) paper to deal with smart healthcare systems. PAP is made up of three primary components: sensor, controller, and medical server. The sensor module detects the data, assigns a dynamic priority to the data packet based on the estimated values, and then delivers it to the controller unit according to the data packet's determined priority. The major goal of this article is to send data from a node to a coordinator node and then to a patient database in a timely and reliable manner (21). Radio waves on the receiving end are used to calculate the (RSSI). RSSI used 128 bits of size for data. Implementation of RSSI is complex and requires high memory. The restricted data density of RSSI-based key generation and agreement is a major problem. Unlike them, the research presents a physical layer-based security strategy in this work that uses physical channel information and eliminates the need for additional hardware (22).

#### 2.1.10 Blockchain

WBAN provides a quick approach to gathering patient data, but they also introduces severe issues, the most important of which is the secure storage of the data obtained. WBAN devices' data storage and data security do not fulfill the demands of WBAN customers. As a result, the (Ren) paper uses a blockchain database to collect data, which increases the data's security. In addition, the research paper solution proposes a blockchain-based storage architecture for WBAN. The blockchain's storage space is limited, and the data it stores is exposed to unwanted access. To address these issues, the article presented a sequential aggregate signature method with a specified verifier (DVSSA), which ensures that a user's data may only be read by the authorized person and protects WBAN users' privacy (16).

#### 2.1.11 Characteristic of the channel

The essential component of many telemedicine applications, such as customized medicine and home-based smartphone apps is a wireless body area network (WBAN) that uses wireless media to offer data transfer services. WBAN is an important field that is used to transmit patients related important information. Because of the wide accessibility of media in WBAN, malicious tapping or tampering attacks can readily occur, stealing personal information or introducing incorrect data. To avoid this type of attack (Zhang) proposed a mechanism that is used to channel characteristic aware privacy protection method for WBAN is suggested to improve user privacy at a relatively low cost and with great flexibility. Tempering attacks, malicious node attacks, and inserting fake data attacks may all be possible as a result of the great accessibility of resources.

#### 2.1.12 Survey scheme

The systematic literature review (SLR) of multiple protection schemes for WBAN is presented in this survey paper. The study came up with a research question to look at the possibilities of multiple attacks while keeping memory constraints in mind. The study used quality valuation to ensure that the schemes were relevant to the research question. Furthermore, the schemes are examined from 2016 to 2020 to concentrate on recent work. Several current systems are investigated in the literature to determine how the protection of sharing patients' healthcare data can be improved. The study degree of confidence and satisfaction required by patients (29). Also examines the protection of various attack scenarios. The efficient transmission of data over a wireless channel may be disrupted by a variety of attacks (29). Existing studies include an overview of data protection in the medical environment, but the research concentrated on data security schemes in WBAN that reduce various attacks to provide the degree of confidence and satisfaction required by patients (29). WBAN is highly beneficial in today's environment, but it faces a variety of issues that must be overcome before it can be used. This (Singh) research considers different systems and protocols for dealing with energy efficiency, security, and privacy in depth. WBAN is a type of Wireless Sensor Network that comprises tiny bio-medical types of equipment known as nodes that are dedicated to guaranteeing continuously patient monitoring based on certain essential criteria. Because of its benefits, including portability, flexibility, and simplicity of patient monitoring, smart healthcare has gotten a lot of attention. WBAN is made up of a variety of heterogeneous devices, thus the amount of data and bandwidth required varies depending on their characteristics (19). First, the research discussed the article title, year, and references in this research Table 1. Then discuss the objective of each paper and also the technique or methodology. Finally, critical analyses were discussed for each of them. Gathered a variety of literary techniques to give us the ability to come up with new ways to defend against attacks that are vulnerable to the schemes. Because of their complex algorithms, the majority of research methods are time and cost-intensive. AES is difficult to implement on software in a way that is both fast. Table 2 compares the security of various schemes in the literature based on eavesdropping (30), denial of service (DoS) (31), malicious nodes (32), and execution time and cost. Several schemes have been proposed in the literature to examine the strengths of these security mechanisms to reduce attacks in the WBAN scenario.

**Table 2:** Comparative analysis of techniques.

**Scheme**	**Time**	**Eavesdropping**	**DOS**	**MN**
Chowdhury et al. (11)	+	+	-	-
Rehman et al. (7)	+	-	+	-
Shanthapriya and Vaithianathan (12)	-	+	-	+
Braham et al. (4)	-	-	-	-
Jabeen et al. (8)	+	-	-	-
Jabeen et al. (8)	-	-	-	+
Mehmood et al. (26)	+	+	-	-
Parvez et al. (10)	+	-	-	-
Roy et al. (15)	+	-	+	-
Anwar et al. (14)	+	+	-	-
Farooq et al. (28)	-	-	+	+
Jabeen et al. (29)	+	+	-	-
Hasan et al. (9)	+	-	+	+
Ren et al. (16)	-	-	-	+
Zhang and Ma (17)	-	+	-	+
Shanmugavadivel et al. (18)	+	-	-	-
Singh and Prasad (19)	+	-	-	-
Soni and Singh (20)	+	-	-	+
Sandhu and Malik (21)	+	+	-	-
Li et al. (22)	-	-	-	-

## 3 Research gap

The research gaps according to the literature review are reported here. In the research Papers (7, 13, 29) uses the AES algorithm and that is a very complex algorithm. AES is a complex and costly algorithm and not suitable for sensor networks. This algorithm is complicated to implement. Encryption is difficult with large key sizes. Furthermore, decrypting data with this algorithm takes a longer time. And also these schemes are affected by DOS and IoT-based attacks. In this research paper (22) RSSI scheme is presented and this algorithm is difficult to implement because it is complex. In research paper (21) PAP(Priority Aware Protocol) as the priority level rises, so does the amount of energy expended. In the paper, Ren et al. (16) DVSSA has proposed it is a time-consuming and complex technique also data tampering attack is possible on it. In paper, Roy et al. (15) SDN(Software Defined Networking) is proposed. This scheme is affected by two attacks and that is DOS, MINA. Vulnerabilities in security, and inconsistency SDESW's flow demands rise as the network becomes more complex. In this research paper (20) LAKA is presented and that is increased energy use.

## 4 Conclusions

The protection of data in WBAN is important and should not be neglected. WBAN is used for gathering the medical conditions of patients and is sent to any portable device that is linked to databases that can store patient details. Because of the critical importance of the health issue, it must be kept hidden from unauthorized persons. In addition to highlighting security and privacy problems, a number of approaches for a WBAN utilizing IoT systems are thoroughly evaluated. Only a few research methodologies are considered viable due to the multifaceted nature of WBAN, and there are some extremely challenging and difficult research methodologies. This literature focuses on various approaches to information security however, only a few are considered to be superior to others in terms of information security. Various current strategies are observed in the literature to understand how the security of patient's health data is upgraded.

## Author contributions

WJ: Formal analysis, Investigation, Methodology, Software, Supervision, Validation, Writing – original draft. AT: Conceptualization, Data curation, Formal analysis, Investigation, Methodology, Software, Writing – original draft. JL: Formal analysis, Funding acquisition, Investigation, Methodology, Project administration, Resources, Supervision, Validation, Visualization, Writing – original draft.
